# Residual Defect Density in Random Disks Deposits

**DOI:** 10.1038/srep12703

**Published:** 2015-08-03

**Authors:** Nikola Topic, Thorsten Pöschel, Jason A. C. Gallas

**Affiliations:** 1 Institute for Multiscale Simulation, Friedrich-Alexander-Universität Erlangen-Nürnberg, Erlangen, Germany; 2 Departamento de Física, Universidade Federal da Paraíba, 58051-970 João Pessoa, Brazil; 3 Instituto de Altos Estudos da Paraíba, Rua Infante Dom Henrique 100-1801, 58039-150 João Pessoa, Brazil

## Abstract

We investigate the residual distribution of structural defects in very tall packings of disks deposited randomly in large channels. By performing simulations involving the sedimentation of up to 50 × 10^9^ particles we find all deposits to consistently show a non-zero residual density of defects obeying a characteristic power-law as a function of the channel width. This remarkable finding corrects the widespread belief that the density of defects should vanish algebraically with growing height. A non-zero residual density of defects implies a type of long-range spatial order in the packing, as opposed to only local ordering. In addition, we find deposits of particles to involve considerably less randomness than generally presumed.

An enticing class of problems, familiar from real-life and from several scientific disciplines, is the class dealing with the so-called *packing problems*, namely with the generic optimization problem of packing as close as possible objects of variegated shapes, with or without spatial confinement^1^,^2^,^3^,^4^,^5^,^6^. Packing problems are of great academic interest and ubiquitous in practical applications, from packing goods into containers to the design of printed circuit boards for electronic devices. The problem is also related to jamming of dynamical granular systems, being subject of a large number of publications, e.g.^7^,^8^,^9^,^10^. Dense packings of particles lead to minimization of material porosity which is relevant, e.g., for sintering of nano-powders used in engineering new materials with superior mechanical properties^11^,^12^,^13^. In computer sciences, to find the optimal solution for a packing is generically NP complete^14^, meaning that practical algorithms must rely on heuristics.

The interest in packing problems can be traced easily back to ancient times. They already received significant attention by a number of eminent scientists, and are well-known to be notoriously difficult, even in the elementary case of spherical particles on an unbounded domain^15^,^16^. For instance, Kepler’s celebrated conjecture of 1611 concerning the densest sphere packing arrangement was only proved in 2005 by Hales, following an approach suggested in 1953 by Tóth^17^. Greater challenges and surprises arise for packing problems in confined volumes. For instance, an unexpected and beautiful result is the discovery that the optimal packing of oblate spheroids is different from the optimal fcc packing of spheres. Although oblate spheroids may be always packed in fcc scheme leading to the same packing fraction as the fcc packing of spheres, it was shown that fcc packing is not optimal for oblate spheroids^18^.

Here, we report a remarkable result obtained while investigating systematically the structure of very large deposits of particles formed by up to 50 × 10^9^ particles, namely for deposits much larger than the ones considered thus far. Our main result concerns the density of defects observed asymptotically in the deposit: Instead of converging to a defect-free packing as believed for quite some time^19^,^20^, we find that, in fact, the asymptotic density of defects invariably displays a nonzero remnant value. In the remainder of the paper we describe how our sedimentation experiments were done and the generic structural organization observed in the sediments.

Following a large *corpus* of literature, we study sediments of monodisperse frictionless hard disks. We simulate an experiment which consists of dropping one-by-one disks of diameter *d* into a vertical channel of width *w* measured in disk diameter *d*. As usual, we use periodic boundary conditions in the horizontal direction. Particles are dropped vertically from initial positions chosen randomly with uniform probability distribution. The action of inertia is assumed to be absent or negligible during the sedimentation process, a situation that can be implemented experimentally, e.g., by working in the presence of an ambient viscous fluid. Absence of inertia allows us to use the familiar ballistic deposition algorithm of Visscher and Bolsterli^21^,^22^,^23^,^24^,^25^, described below, in *Methods*. This algorithm is of fundamental importance to be able to construct very large deposits. The algorithm is invaluable for obtaining specific but reliable results for very large systems, systems whose dynamics is computationally intractable with other techniques like, for instance, molecular dynamics.

## Results

**Figure 1:**
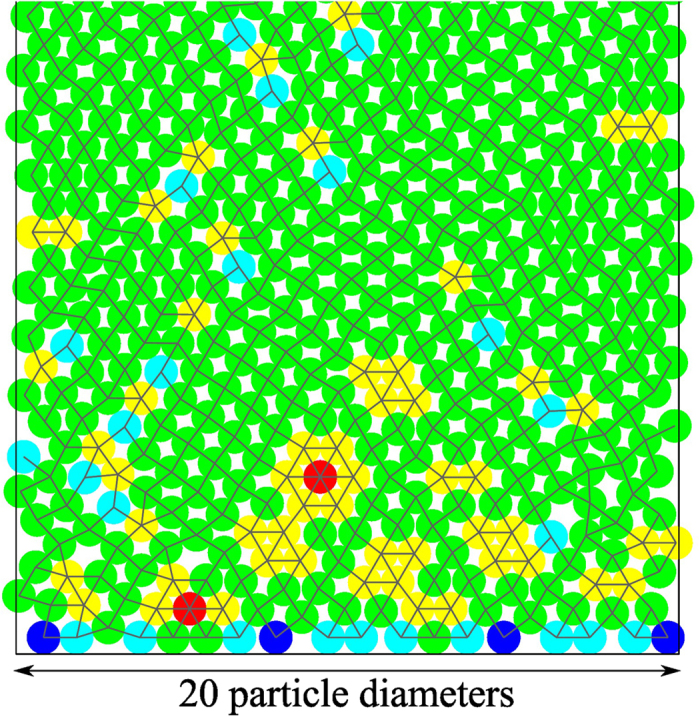
A representative packing illustrating with colors the distribution of defects. The lateral vertical lines mark the limits of the periodic boundaries of the box. Particles with two contacts are shown in blue, with three in cyan, four in green, five in yellow, and six in red. Bonds between particles are shown as line segments connecting their centers. Bonds across opposite sides of the periodic domain are not drawn. Note the presence of voids (small white empty spaces).

Figure 1 shows a typical example of the packing structure generally observed at the bottom of the channel, here for a width of *w *= 20 particle diameters. As indicated in the figure caption, the color of the particles encode their coordination number. The average coordination number of a two-dimensional stable packing is slightly above four, disregarding particles touching the ground and particles located at the free surface. This follows from the fact that each particle added to the sediment creates two new contacts, three in rare cases, when finding its stable position. Following common practice, any particle having other than four contacts is considered to be a *defect*. This definition is equivalent to other ones based on counting polygons made of neighboring bonds different from a rhombus^20^. Thus, all particles not in green represent defects of the sediment. The existence or not of contacts (and defects) depends of course of the ordering of the sequential dropping.

There are two sources of randomness acting in the formation of the deposit. First, the randomness enforced by the bottom layer, where particles are immobilized either after direct vertical fall to the ground or, after hitting a particle already at the bottom, by rolling over it until also hitting the ground, where both particles stay in contact. Since the dropping process is homogeneously distributed along the channel, particles on the bottom layer are not equally distributed. The bottom layer in Fig. 1 contains only 17 particles, out of 20 that could be fitted side-by-side in the channel. The pile grows essentially in a row-by-row manner, with the bottom layer reflecting the specific random sequence of the initial dropping process. The particular distribution of particles in the bottom layer is very important for the subsequent growth process since it defines the local minima which are available for the particles in all subsequent layers.

The second randomness in sediment formation arises from the specific ordering characteristic of the sequential dropping of successive layers. Although the local minima are all fixed by the bottom layer, the sequential order of the subsequent depositions is the key for shaping the final sediment. Its structure arises from the interplay of two mechanism: the initial definition of the local stability minima by the bottom layer combined with the sequential order in which such minima are filled by falling particles. The infinite tree of possible minima available to form specific piles is fixed by the bottom layer but the specific pile effectively realized is selected step-by-step by the whole sequential deposition process. Minute changes in the initial creation and/or the subsequent selection of the chain of minima will drastically change the structure of the final deposit. The selection of the minima is a finite and deterministic branching process.

**Figure 2:**
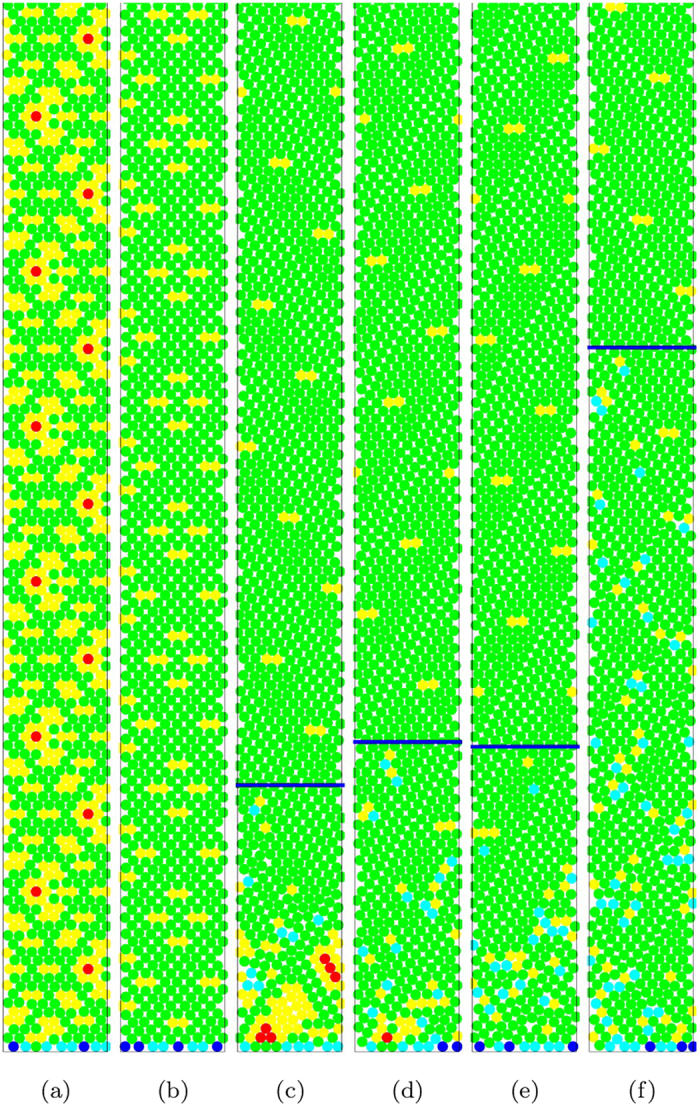
Typical residual defect distributions observed for six representative packings, illustrating the asymptotic periodic structures as well as the distinct transient phases preceding them. Here, the channel width is *w *= 10 particle diameters and the height *h* is about 10*w*. For small widths, transients can be rather small. The structure of voids (white empty spaces) is also visible. When not immediate, the onset of periodicity is indicated by a horizontal line.

Figure 2 shows some examples of the complete packing structure normally found along the channel. They illustrate the distribution of defects for small width channels, for better visibility. From these examples one sees that, essentially, the amount of defects decays rapidly with height. However, we consistently find the average density of defects to be non-zero for all deposits investigated. In other words, we find the remnant density of defects to be an integral part of the asymptotic periodic part of the packings.

We observed two basic types of remnant defect patterns. In the first, Fig. 2(a,b), the whole packing is periodic from the start, including the first layer. In the second, exemplified by Fig. 2(c–f), defects appear as isolated structures and the packing becomes periodic only after an initial transient growth that can be relatively long [Fig. 2(f)]. In this second type, we included a fraction of 2% to 3% of piles which display *almost zero* initial transients. Figure 2 shows defects to be regularly spread along the channel and to be traceable by performing simple translations along the channel. Asymptotically, the channel is tiled by periodic patterns, mosaics, which, except for some exceptional very symmetrical initial conditions, always contain defects embedded in them.

**Figure 3:**
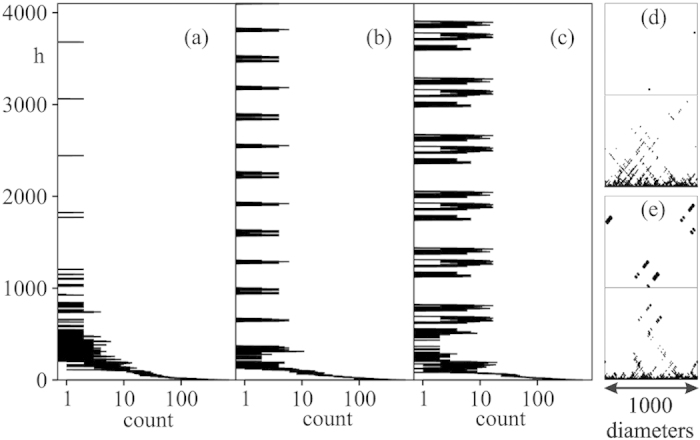
(**a–c**) Number of defects (count) with height, *h*, for three packing realizations in a channel of width *w *= 1000. **(d)** Spatial distribution of defects for the packing from figure (**a**), for 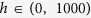 (bottom) and *h *= (3000, 4000) (top). (**e**) Same as (**d**) except that packing corresponds to figure (**c**).

Figure 3 depicts the density of defects as a function of height for a moderately large sediment of width *w *= 1000. The counts at height *h* show the number of defective discs cut by a horizontal line at height *h*. The defect counts decay rapidly with height and show periodic repetitions after growing for about 1 to 2 channel widths. In all three cases there is a remnant defect density. We have plotted the defect counts up to height of 20 channel widths, and the periodic patterns persisted. The defect counts vary strongly between runs. Figure 3(d,e) shows the spatial distribution of defects.

**Figure 4:**
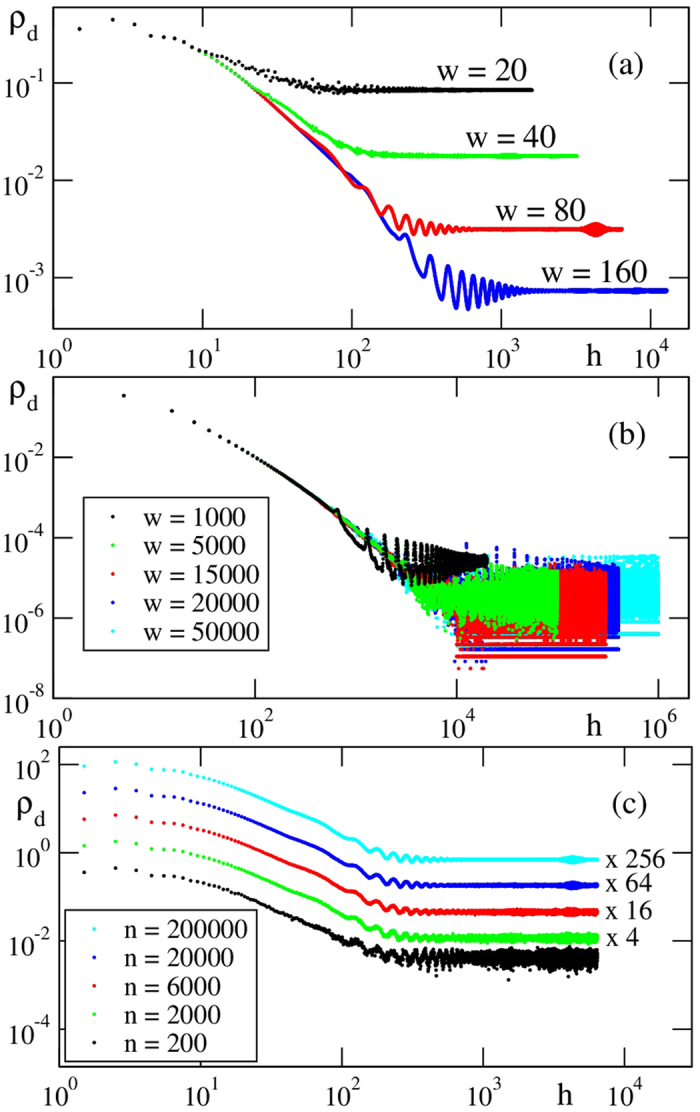
Density of defects *ρ*_*d*_ as a function of height *h* for various channel widths, *w*. (**a**) For each channel width the average is over 5 × 10^5^ sediments. (**b**) The densities of defects are averages over 12000, 2400, 120, 60 and 10 sediments, for channel widths *w* = 1000, 5000, 15000, 20000 and 50000, respectively. (**c**) The approach to the asymptotic density does not depend of the sample size used for averaging. Here, *w* = 80.

Figure 4 records the asymptotic density of defects measured for packings ranging from medium, 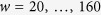, up to very large widths, 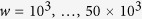, and heights. Here, the defect density is defined as 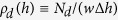, where *N*_*d*_ is the number of defects found in the interval [*h *− Δ*h/*2, *h *+ Δ*h/*2] (linear binning). Each curve in Fig. 4(a) results from an average over 5 × 10^5^ sediments. For the larger systems, Fig. 4(b), the level of averaging (given in the caption) is smaller leading to larger noise levels. Fitting a power law to *ρ*_*d*_(*h*) = *ρ*_*d*_ ~ *h*^*α*^ in the range 5 ≤ ln *h *≤ 7 we obtain *α *= −1.86 ± 0.06. Using logarithmic binning (i.e., binning ln *ρ*_*d*_ as a function of ln *h*), we obtain *α *= −1.91 ± 0.09. The small oscillations of *ρ*_*d*_(*h*) just before reaching the asymptotic level seen in Fig. 4 are Moiré-like artefacts produced by superposing (averaging) over a large number of periodic patterns with different period due to distinct asymptotic periodic structures of the sediments. Within the statistical error, our results agree well with the scaling found by Meakin and Jullien^20^, who found *ρ*_*d*_ ~ *h*^*α*^ where *α *= −1.92 ± 0.10.

**Figure 5:**
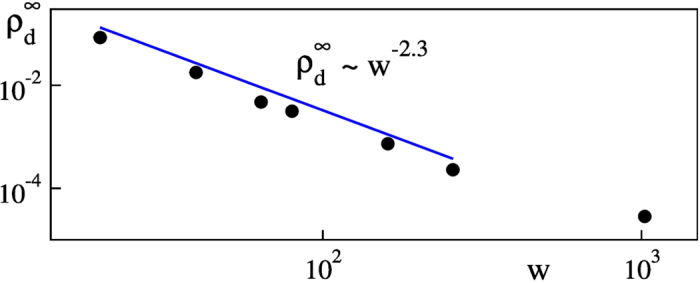
Asymptotic density of defects, 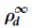 as a function of the channel width, *w.*

Figure 5 shows the asymptotic density of defects, 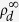, plotted as a function of the channel width, *w*. The asymptotic density of defects is an average of defect densities between two heights inside of the plateau regions. As seen from this figure, it scales as *w*^−2.3^. Thus, we find that the density of defects for large height converges asymptotically to approximately constant values, 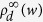.

In the literature, there is a widespread belief that the asymptotic structure of depositions is defect-free, a result that we could trace back to an interesting paper by Meakin and Jullien^20^. They investigated widths up to *w* = 16384 and (when measuring defect density) heights up to 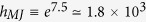 concluding that, in the limit of large height, the defect density *ρ*_*d*_ vanishes, a result conflicting with our present findings. For *w *= 16384 we observe the transient regime to extend up to heights of about *h* = 10^5^. What is the origin of this discrepancy? First, we remark that *h*/*h*_*MJ*_ ≈ 56 and that, accordingly, their computations were done still during the transient regime. Second, from Fig. 3(a–c) we see that it is not uncommon for large channels to show no defects over extended portions of the sediments, corresponding to (large) transients needed for the spatial re-arrangements to allow defects to recur periodically. Third, the absence of defects over relatively large portions of the sediment combined with the large investment of time then required may have induced one to stop simultations too early. Thus, it is possible that the discrepancy may be related to the sparse distribution of defects in large sediments.

From the statistical expressions obtained here for the width and height of the channel it is possible to estimate suitable height/width thresholds so as to avoid the aforementioned discrepancy for any experiments involving large sediments. To this end, we equate 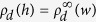, where 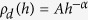 and 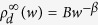. This gives *h*/*w *= (*A*/*B*)^*1/α*^*w*^β/α−1^ ≈ *Cw*^0.2^ where *A*, *B*, *C* are constants and where data from Fig. 4 was used for the exponents. Thus, the height/width discrepancy threshold depends not much on the channel width, being essentially constant between *w *= 10^3^ and *w *= 20 × 10^3^. For *w *= 15000 we estimate *h*/*w *≈ 1. This means that *h*_*MJ*_/16384 ≈ 0.11 while for the value used in our present simulations the corresponding ratio is 10^5^/16384 = 6.10, safely above the *h*/*w *≈ 1 estimated threshold.

## Discussion

The simulations reported here revealed that the residual distribution of structural defects in tall packings of disks deposited randomly in large channels consistently shows a non-zero residual density of defects obeying a characteristic power-law. This implies a type of long-range spatial order in the packing, as opposed to only local ordering. This type of effect might be detectable in a structure factor or pair correlation function calculation, where a pair correlation function would presumably decay with a power law tail rather than exponential.

Our systematic study of very large piles of particles provided new insight about the random processes underlying the sequential deposition. Filling the basal plane is a *random* process, but all subsequent particle depositions are completely *discrete and deterministic*. Filling the basal plane of the pile automatically fixes a *unique discrete tree of local minima* that provides stable refuge for all subsequent incoming particles dropped onto the pile. Such local minima have wide *basins of attraction*, namely large dropping intervals from which particles are attracted to them, being thus virtually insensitive to residual imperfections of real-life random-number generators. Discrete trees remain robust even for non-perfect generators. After filling the basal plane, the only freedom still left is the freedom to select the specific *ordering* of filling the *discrete* set of local minima. In other words, the remaining freedom amounts to selecting the branching path into a tree of prescribed and well-defined minima, a process that involves a finite set of possibilities.

These findings imply the final packing to be ultimately quasi-periodic, i.e. any temporal behavior must be a discrete superposition of finitely or countably many Fourier components with discrete frequencies. In the ergodic theory of classical dynamical systems, this quasi-periodic dynamics corresponds to the limiting case of integrable or ordered motion while truly chaotic motion requires continuous Fourier spectrum^26^. Thus, piles of particles involve far less dynamic randomness^27^ than generally presumed. It should be interesting to compare our results with similar ones obtained by using different deposition algorithm. However, due to the exceptionally large sizes of our piles, such comparisons may be not completely trivial to perform.

## Methods

The ballistic deposition algorithm of Visscher-Bolsterli is not complicated and works as follows^21^,^22^,^23^,^24^,^25^. Dropped particles move either straight down unimpeded to the basal plane or follow the trajectory of steepest descent if touching the surface of the sediment before they can reach the basal plane, or rest on two particles. Particles keep moving until they reach a local minimum, i.e. a stable position of equilibrium. Upon reaching a stable position the particle is no longer allowed to move, being *glued* (permanently added) to the growing sediment. A new particle is only dropped when the previous one has finished its steepest descent motion. To characterize local equilibrium, the Visscher-Bolsterli algorithm uses a criterion to detect that particles are in contact. Here, we assume particles to be in contact when their surfaces are closer than *ξ* = 10^−10^ particle diameters. We verified that the final configurations remain unchanged for contact distances *ξ* in the interval 10^−6^ < *ξ *< 10^−12^.

## Additional Information

**How to cite this article**: Topic, N. *et al*. Residual Defect Density in Random Disks Deposits. *Sci. Rep*. **5**, 12703; doi: 10.1038/srep12703 (2015).
